# Correlating topological indices with physicochemical properties in 15 polycyclic aromatic hydrocarbons

**DOI:** 10.1038/s41598-025-29525-x

**Published:** 2025-11-26

**Authors:** Zeming Huang, Jing Zhao, Bangbang Jin, Jiayi Cao, Yu Yang, Zhen Li

**Affiliations:** 1 School of Software, Pingdingshan University, Pingdingshan, 467000 China; 2 School of Computer Technology, Henan Quality Institute, Pingdingshan, 467000 China; 3 China National Institute of Standardization, Beijing, 100191 China; 4 Henan International Joint Laboratory for Multidimensional Topology and Carcinogenic Characteristics Analysis of Atmospheric Particulate Matter PM2.5, Pingdingshan, China; 5 School of Automation, Northwestern Polytechnical University, Xian, 710072 China

**Keywords:** Distance-based topological indices, Degree-based topological indices, Physicochemical properties, $PM_{2.5}$, Regression models, Chemistry, Environmental sciences

## Abstract

$$PM_{2.5}$$, a major air pollutant, often carries toxic components such as polycyclic aromatic hydrocarbons (PAHs), which pose potential health risks. This study investigated the relationships between five physicochemical properties-boiling point, molecular mass, enthalpy of vaporization, molar volume, and surface tension-of 15 PAHs and seven molecular topological indices (including the Wiener, Hyper-Wiener, Distance-degree, and Gutman indices) using regression analysis. Results indicated that the selected topological indices were strongly correlated with the properties of PAHs, with correlation coefficients generally exceeding 0.7. In particular, quadratic regression models based on the Distance-degree and Gutman indices showed a very high correlation with enthalpy of vaporization ($R = 0.998$). This work provides quantitative insights into the structure–property relationships of PAHs and may support further theoretical developments in the risk assessment and source control of $$PM_{2.5}$$-bound PAHs.

## Introduction

Fine particulate matter ($PM_{2.5}$), defined as particles with an aerodynamic diameter of 2.5 $\upmu$m or less, is a major component of air pollution. Owing to its small size and capacity for long-range transport, $PM_{2.5}$ can penetrate deeply into and deposit within the human respiratory system. This leads to various health risks, including respiratory diseases and cardiovascular impairment, in addition to exerting considerable influence on regional climate and visibility^1^.

With the ongoing acceleration of global urbanization and industrialization, ambient $PM_{2.5}$ concentrations are continually increasing. Its complex chemical composition, which includes polycyclic aromatic hydrocarbons (PAHs), heavy metals, and organic carbon, enhances its toxicity through synergistic interactions among its physicochemical constituents^2^. Among these, PAHs are widely acknowledged as key contributors to the toxicity of $PM_{2.5}$. PAHs constitute a large group of hydrocarbons composed of two or more fused benzene rings, encompassing nearly ten thousand homologues^3^.

The research history of PAHs can be traced back to the rise of the coal tar industry in the 19th century. In 1933, Cook et al.^4^ isolated benzo[a]pyrene (BaP) and established its potent carcinogenic effects. By the mid-20th century, pollution disasters such as the London Smog event revealed a positive correlation between PAH concentrations in $PM_{2.5}$ and mortality from respiratory diseases^5^, prompting the scientific community to re-evaluate their environmental toxicity attributes. Since being listed as priority pollutants by the U.S. Environmental Protection Agency (USEPA) in 1976, 16 PAHs have been recognized by the World Health Organization (WHO) as a major public health concern due to their persistent and toxic nature. This recognition has led to their mandatory monitoring in over 50 countries and regions, including the European Union and China. Consequently, research on the structure–property relationship mechanisms of PAHs and the control of their exposure risks has become a core frontier topic in environmental science and public health.

From a molecular structure perspective, PAHs can be classified into non-fused ring types and fused ring types. Fused-ring PAHs, due to their significant bioaccumulation potential and carcinogenicity, have become the focus of research^6^. Variations in the number of benzene rings (2–7 rings) and fusion patterns lead to significant differences in toxicity gradients and physicochemical properties^7^. This study focuses on the 15 fused-ring PAHs designated as priority pollutants by the USEPA (representative compounds include benzo[a]pyrene, phenanthrene, fluoranthene, etc.). Since acenaphthene and acenaphthylene share an effectively identical molecular framework and virtually identical physicochemical properties, we selected one of them, acenaphthene, for our experimental investigation. It employs graph theory methods to establish quantitative structure-property relationship (QSPR) models linking molecular topological features to physicochemical properties, enabling analysis and prediction.

Graph theory deciphers molecular structures by abstracting them into graphs, where atoms and bonds are represented as vertices and edges. This framework enables the use of topological indices, which are numerical descriptors that characterize molecular topology. These indices are indispensable for quantifying compound activity, predicting properties, and identifying isomers. Consequently, applying graph-theoretic indices to study the structure-activity relationships of PAHs is highly significant and has spurred extensive interdisciplinary research^8–10^.

## Related works

In 1947, Harry Wiener pioneered the proposition that the boiling points and other physical properties of organic compounds are primarily determined by the number, type, and topological configuration of atoms within the molecule. For a series of isomers, where the number and type of atoms remain constant, differences in physical properties can be entirely attributed to topological variations in structural interconnectivity^11^. This study first validated the feasibility of using topological descriptors to predict physicochemical properties, laying the theoretical foundation for topological Quantitative Structure Activity Relationships (QSAR)^12–14^.

The establishment of the Hansch equation in 1962 marked the birth of the modern QSAR paradigm^15^. Shortly thereafter, a critical breakthrough was achieved in 1964 with the Free-Wilson approach^16^, which introduced a structure fragmentation method. This feature extraction technique, reliant solely on two-dimensional structure, not only built a crucial bridge between chemical topology and physicochemical/biological properties but also directly propelled the maturation of the 2D-QSAR paradigm. By converting topological structures into computable numerical features, it liberated property prediction from dependence on experimental parameters, thereby laying the groundwork for efficient molecular design.

With the maturation of classical QSAR paradigms (the Hansch equation and the Free-Wilson method), researchers began exploring quantitative predictive models that rely solely on the two-dimensional topological structure of molecules, namely 2D-QSAR. The core innovation of this approach lies in abandoning the dependence on experimental physicochemical parameters and instead extracting mathematical descriptors from molecular graph theory. Wiener pioneered the use of the path number, later formalized as the Wiener Index, to quantify molecular branching, revealing an inverse correlation between the boiling points of alkanes and topological complexity. Randić further advanced the field by developing the Molecular Connectivity Index, which predicts properties like hydrophobicity based on atomic adjacency relationships, thereby solidifying the mathematical foundation of topological descriptors^17^. The core advantages of 2D-QSAR are its computational efficiency and structural generalizability: it transforms molecular structural features into quantifiable physicochemical parameters, allowing for the rapid establishment of structure property relationships with limited resources, making it highly suitable for large-scale virtual screening.

In 2004, the OECD enacted five key validation principles (a defined endpoint, an unambiguous algorithm, a defined domain of applicability, appropriate validation metrics, and a mechanistic interpretation, if possible), mandating a major global consolidation of descriptor systems. The *Handbook of Molecular Descriptors* by Todeschini and Consonni is revered as the “computational bible” of the field^18^. This manual for the first time incorporated topological parameters like the Wiener index and Randić connectivity index, geometric features like the molecular moment of inertia, and quantum chemical descriptors like the Fukui function into a unified classification framework, constructing a universal translation framework from structural features to mathematically computable features.

In 2024, Muhammad Waheed Rasheed et al.^19^ investigated the properties of ocular infection drugs by utilizing nine vertex-degree-based topological indices and applying linear regression models. They introduced two novel indices: the “First Revised Randić Index” and the “Second Revised Randić Index.” Their experimental analysis validated correlations between these indices and several physicochemical properties of the drugs, including molecular weight, boiling point, enthalpy, flash point, molar refraction, and molar volume. The results demonstrated the effectiveness and accuracy of employing degree-based topological indices within linear regression frameworks for estimating drug properties. This work strongly underscores the significant correlation between topological indices and the physicochemical attributes of molecules.

In 2025, Priyanka et al.^20^ demonstrated significant correlations between computed topological indices and various physicochemical properties of molecules, underscoring the effectiveness of these indices in modeling and predicting molecular behavior. The study focused on degree-based topological indices, such as the Wiener index, Randić index, and Zagreb indices, and evaluated their predictive power for the physicochemical properties of PAHs. The researchers employed regression analysis to correlate these indices with experimentally determined properties, including boiling point, entropy, acentric factor, octanol-water partition coefficient, enthalpy of formation, and Ková retention index. The results indicated that certain topological indices exhibited strong correlations with specific molecular properties, suggesting their potential as reliable predictors in Quantitative Structure Property Relationship (QSPR) studies.

In recent years, graph-based topological indices have emerged as a major research focus in QSPR/QSAR studies. By encoding the structural characteristics of compounds into numerical descriptors, these indices establish a quantitative bridge between molecular topology and its macroscopic properties or biological activities, thereby paving the way for the effective prediction and discovery of specific compounds.

## Topological indices and the regression models

Next, we introduce the necessary notations and definitions of the topological indices used in this study.

Let $G = (V(G), E(G); f, g)$ be a weighted graph of order $n$ and size $m$, where $V(G) = \{v_1, v_2, \dots , v_n\}$ is the vertex set and $E(G) = \{e_1, e_2, \dots , e_m\}$ is the edge set. The distance between vertices $u$ and $v$ is denoted by $d(u, v)$, and the degree of a vertex $v \in V(G)$ is written as $\deg (v)$.

The Wiener index^11^, a pioneering topological index in molecular topology, has found extensive applications in chemical graph theory and network science due to its correlations with a wide range of molecular properties ranging from boiling point and melting point to refractive index and network characteristics such as transmission efficiency and robustness. It is defined as follows:

1 $$\begin{aligned} W(G) = \sum _{\{u,v\} \subseteq V(G)} d(u, v). \end{aligned}$$

The Hyper-Wiener index^21^ was introduced by Milan Randić in 1993 as a generalization of the Wiener index. It is defined as:

2 $$\begin{aligned} HW(G) = \frac{1}{2} \sum _{\{u,v\} \subseteq V(G)} \left( d(u,v) + d^2(u,v) \right) . \end{aligned}$$

The Distance-degree index^22^ integrates vertex degrees with shortest path distances, providing a robust metric for characterizing network topology. As such, it serves as an effective tool for assessing and optimizing structural connectivity and transmission efficiency, with broad applications spanning chemical graph theory, network design, and social network analysis. The index is defined as:

3 $$\begin{aligned} DD(G) = \sum _{\{u,v\} \subseteq V(G)} \left( \deg (u) + \deg (v) \right) d(u,v). \end{aligned}$$

The Gutman index^23^,proposed by Gutman in 1994, is a Schultz-type molecular topological index and a notable variant of the widely studied Wiener index. It is defined as follows:

4 $$\begin{aligned} Gut(G) = \sum _{\{u,v\} \subseteq V(G)} \left( \deg (u) \times \deg (v) \right) d(u,v). \end{aligned}$$

The Harary index was introduced independently by Ivanciuc and Plavšić in 1993 in honor of Professor Frank Harary, the father of modern graph theory^24^. It is defined as follows:

5 $$\begin{aligned} H(G) = \sum _{\{u,v\} \subseteq V(G)} \frac{1}{d(u,v)}. \end{aligned}$$

The Additively Weighted Harary index^25^ is a generalization of the classical Harary index, offering a more nuanced assessment of network connectivity by accounting for differential weighting of connections. This enhanced capability makes it particularly valuable in chemical graph theory, network optimization, and the analysis of systems with heterogenous bond strengths or interaction priorities. Its definition is as follows:

6 $$\begin{aligned} H_A(G) = \sum _{\{u,v\} \subseteq V(G)} \frac{\deg (u) + \deg (v)}{d(u,v)}. \end{aligned}$$

The Multiplicative Weighted Harary Index^26^ differentiates itself from its additive counterpart by multiplying, rather than summing, the vertex-pair distance weights. This approach yields a more nuanced modeling of interaction strength effects, thereby improving optimization applications in drug design, materials science, and network engineering. Its definition is as follows:

7 $$\begin{aligned} H_M(G) = \sum _{\{u,v\} \subseteq V(G)} \frac{\deg (u) \times \deg (v)}{d(u,v)}. \end{aligned}$$

**Fig. 1:**
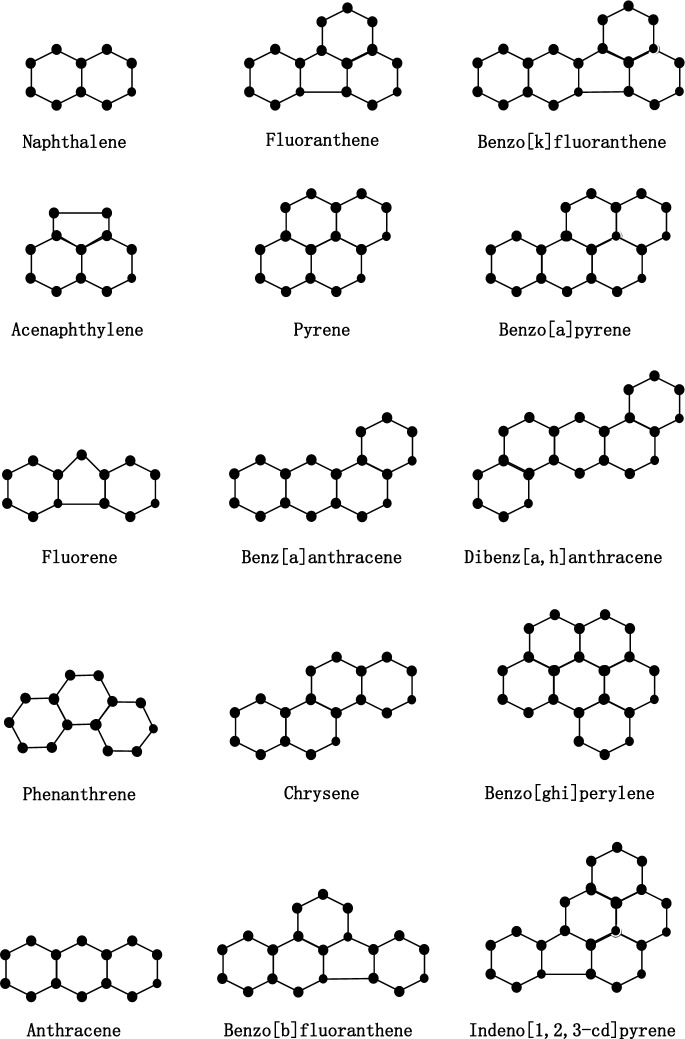
Chemical structures of 15 PAHs.

In this section, we calculate seven topological indices based on degree of vertices and distance between each pair of vertices of 15 PAHs molecules. The 15 PAHs molecules that we consider are: Naphthalene, Acenaphthylene (ACY), Acenaphthylene (ACE), Fluorene, Phenanthrene, Anthracene, Fluoranthene, Pyrene, Benz[a]anthracene, Chrysene, Benzo[b]fluoranthene, Benzo[k]fluoranthene, Benzo[a]pyrene, Dibenz[a,h]anthracene, Benzo[ghi]perylene and Indeno[1,2,3-cd]pyrene. The molecular structures of these 15 PAHs are depicted in Fig. 1.

The edge partition of Naphthalene, based on the degrees of the end vertices of each edge and their respective frequencies, is presented in Table 1. Using this edge partition and the corresponding definitions, the values of the topological indices are calculated as follows.

**Table 1:** Distance and degree-pair frequency distribution for Naphthalene.

Distance *d*(*n*, *G*)	Degree pair $(d_u, d_v)$	Frequency
*d*(1, *G*)	(2,2)	6
*d*(1, *G*)	(2,3)	4
*d*(1, *G*)	(3,3)	1
*d*(2, *G*)	(2,2)	6
*d*(2, *G*)	(2,3)	8
*d*(3, *G*)	(2,2)	8
*d*(3, *G*)	(2,3)	4
*d*(4, *G*)	(2,2)	6
*d*(5, *G*)	(2,2)	2

$$\begin{aligned} \begin{aligned} W(\text {Naphthalene})&=\sum _{\{u,v\} \subseteq V(G)} d(u, v)\\&=11(1) + 14(2) + 12(3) + 6(4) + 2(5)=109, \\ HW(Naphthalene)&= \frac{1}{2} \sum _{\{u,v\} \subseteq V(G)} \left( d(u,v) + d^2(u,v) \right) \\&=\frac{1}{2}(109) + \frac{1}{2}[11(1)^2 + 14(2)^2 + 12(3)^2 + 6(4)^2 + 2(5)^2]=215, \\ DD(Naphthalene)&= \sum _{\{u,v\} \subseteq V(G)} \left( \deg (u) + \deg (v) \right) d(u,v)\\&=1(6)(2+2)+1(4)(2+3)+1(1)(3+3)+2(6)(2+2)+2(8)(2+3)\\&+3(8)(2+2)+3(4)(2+3)+4(6)(2+2)+5(2)(2+2)=470, \\ Gut(Naphthalene)&= \sum _{\{u,v\} \subseteq V(G)} \left( \deg (u) \times \deg (v) \right) d(u,v)\\&=1(6)(2*2)+1(4)(2*3)+1(1)(3*3)+2(6)(2*2)+2(8)(2*3)\\&+3(8)(2*2)+3(4)(2*3)+4(6)(2*2)+5(2)(2*2)=505, \\ H(Naphthalene)&= \sum _{\{u,v\} \subseteq V(G)} \frac{1}{d(u,v)}\\&=\frac{11}{1}+\frac{14}{2}+\frac{12}{3}+\frac{6}{4}+\frac{2}{5}=23.9. \\ H_A(Naphthalene)&= \sum _{\{u,v\} \subseteq V(G)} \frac{\deg (u) + \deg (v)}{d(u,v)}\\&=\frac{(6)(2+2)}{1}+\frac{(4)(2+3)}{1}+\frac{(1)(3+3)}{1}+\frac{(6)(2+2)}{2}+\frac{(8)(2+3)}{2}\\&+\frac{(8)(2+2)}{3}+\frac{(4)(2+3)}{3}+\frac{(6)(2+2)}{4}+\frac{(2)(2+2)}{5}=106.9333, \\ H_M(Naphthalene)&= \sum _{\{u,v\} \subseteq V(G)} \frac{\deg (u) \times \deg (v)}{d(u,v)}\\&=\frac{(6)(2*2)}{1}+\frac{(4)(2*3)}{1}+\frac{(1)(3*3)}{1}+\frac{(6)(2*2)}{2}+\frac{(8)(2*3)}{2}\\&+\frac{(8)(2*2)}{3}+\frac{(4)(2*3)}{3}+\frac{(6)(2*2)}{4}+\frac{(2)(2*2)}{5}=119.2667. \end{aligned} \end{aligned}$$

The topological indices of the other 15 PAHs can be computed in a similar manner. The calculated values are presented in Table  2.

**Table 2:** Topological indices of the 15 PAHs.

Structures	*W*(*G*)	*HW*(*G*)	*DD*(*G*)	*Gut*(*G*)	*H*(*G*)	$H_A(G)$	$H_M(G)$
Naphthalene	109	215	470	505	23.9	106.9333	119.2667
Acenaphthylene	166	332	756	857	33.4	159.1	189.1
Fluorene	219	490	984	1101	36.9833	174.4333	205.4333
Phenanthrene	271	636	1208	1342	41.1429	192.1381	224.0381
Anthracene	279	680	1248	1392	40.7857	189.9429	220.4095
Fluoranthene	364	853	1678	1926	52.4667	255.4333	311.1667
Pyrene	362	845	1676	1933	52.6095	255.4048	309.7048
Benz[a]anthracene	553	1557	2522	2869	60.3635	287.473	341.5254
Chrysene	545	1513	2482	2819	60.7206	289.6683	345.154
Benzo[b]fluoranthene	676	1831	3151	3660	73.6679	362.5048	446.3381
Benzo[k]fluoranthene	698	1970	3264	3805	72.931	358.0429	439.2619
Benzo[a]pyrene	680	1868	3182	3712	73.6802	361.6492	443.5611
Dibenz[a,h]anthracene	971	3162	4492	5186	81.7417	394.3676	474.8446
Benzo[ghi]perylene	815	2148	3894	4637	88.0429	442.3714	555.9381
Indeno[1,2,3-cd]pyrene	845	2338	4038	4810	87.0226	437.0429	548.8952

By searching in the ChemSpider website (https://www.chemspider.com/),NIST Chemistry WebBook (https://webbook.nist.gov/chemistry/) and referencing previously published papers, we obtained the five physicochemical properties of 15 PAHs: boiling point (BP), molecular weight (MW), enthalpy of vaporization (EV), molar volume (MV), and surface tension, as shown in Table  3. Due to the lack of reported boiling point and enthalpy of vaporization data for Benzo[ghi]perylene and Indeno[1,2,3-cd]pyrene in common databases (e.g., ChemSpider, NIST WebBook), regression models for these properties were trained using data from the remaining 14 compounds.

**Table 3:** Properties of 15 PAHs.

Properties	Boiling point (BP)	Molecular weight (MW)	Enthalpy of vaporization (EV)	Molar volume (MV)	Surface tension (ST)
Units	$^{\circ }$C at 760 mmHg	g/mol	kJ/mol	$\hbox {cm}^{3}$	dyn/cm
Naphthalene	218	128.17	54.6	123.5	40.2
Acenaphthylene	280	152.19	64.6	128.1	54.7
Fluorene	298	166.22	72.4	148.3	46.2
Phenanthrene	340	178.23	78.7	157.6	47.9
Anthracene	340	178.23	78.5	157.6	47.9
Fluoranthene	384	202.25	86.8	161.9	59.4
Pyrene	393	202.25	92.4	161.9	59.4
Benz[a]anthracene	437.6	228.29	105.8	191.7	53.5
Chrysene	448	228.29	106.2	191.7	53.5
Benzo[b]fluoranthene	481	252.31	116.8	196	63.4
Benzo[k]fluoranthene	480	252.31	117.4	196	63.4
Benzo[a]pyrene	495	252.31	117.8	196	63.4
Dibenz[a,h]anthracene	524	278.35	131.1	225.8	57.7
Benzo[ghi]perylene	/	276.33	128.9	200.4	74.2
Indeno[1,2,3-cd]pyrene	536	276.33	/	200.4	74.2

## Regression models

In this study, we developed linear, quadratic, and logarithmic regression models to explore the relationships between the seven topological indices (TIs) and the five physicochemical properties of the 15 PAHs. To determine the most appropriate model for each TI-property pair, we employed a model selection strategy based on statistical goodness-of-fit. For a given property and TI, we compared the three model types and prioritized the one with the highest coefficient of determination ($R^2$), while also ensuring the overall model significance via the *F*-statistic ($p < 0.05$). This approach allowed us to identify the model that best explains the variance in the physicochemical property using the topological index. The detailed statistical parameters (*R*, $R^2$, *F*, *p*-value) for all models are summarized in Section 5 (Tables 4, 5, 6, 7, 8, 9, 10), enabling a direct comparison.

In this section, we develop linear, quadratic, and logarithmic regression models to predict the five physicochemical properties of 15 PAHs. Let $\mathbb {P}$ denote any of the five physical properties of PAHs and *TI* represent the topological index value. Linear regression is employed to predict the value of the dependent variable ($\mathbb {P}$) based on the independent variable (*TI*). This approach can be extended to a quadratic model, which typically requires a more extensive dataset, and a logarithmic regression model, which applies logarithmic transformations to linearize the relationship between variables. These modeling techniques facilitate the establishment of more accurate and meaningful relationships between the variables. The significance of the associations between all topological indices (TI) and the physicochemical properties of PAHs is evaluated using these three models. The equations for these models are defined as follows:

$$\begin{aligned} \mathbb {P}= & a+b(TI), \\ \mathbb {P}= & a+b(TI)+c(TI)^2, \\ \mathbb {P}= & a+b\ln (TI), \end{aligned}$$

where $\mathbb {P}$ represents the property of the molecular structure, *a* is a constant, *b* and *c* are the regression coefficients, and *TI* denotes the topological index. The five physicochemical properties investigated for 15 PAHs are: boiling point (BP) in $^{\circ }$C at 760 mmHg, molecular weight (MW) in g/mol, enthalpy of vaporization (EV) in kJ/mol, molar volume (MV) in $\hbox {cm}^{3}$, and surface tension in dyn/cm. These properties are analyzed using the seven newly defined *TI*s mentioned previously. The results of the linear, quadratic, and logarithmic regression models for each degree-based topological index are presented below. Through experimentation and computation, we obtain the numerical values for the following models.

### Wiener Index *W*(*G*)

*Linear Models:*

$$\text {BP} = 233.484 + 0.354 \cdot W(G)$$

$$\text {MW} = 128.344 + 0.176 \cdot W(G)$$

$$\text {EV} = 52.743 + 0.091 \cdot W(G)$$

$$\text {MV} = 122.658 + 0.106 \cdot W(G)$$

$$\text {ST} = 43.240 + 0.028 \cdot W(G)$$

*Quadratic Models:*

$$\text {BP} = 158.488 + 0.740 \cdot W(G) -0.0004 \cdot W(G)^2$$

$$\text {MW} = 101.657 + 0.313 \cdot W(G) -0.0001 \cdot W(G)^2$$

$$\text {EV} = 39.389 + 0.161 \cdot W(G) -0.0001 \cdot W(G)^2$$

$$\text {MV} = 106.943 + 0.186 \cdot W(G) -0.0001 \cdot W(G)^2$$

$$\text {ST} = 37.383 + 0.058 \cdot W(G) -0.0000 \cdot W(G)^2$$

*Logarithmic Models:*

$$\text {BP} = -491.673 + 149.200 \ln (W(G))$$

$$\text {MW} = -227.300 + 73.415 \ln (W(G))$$

$$\text {EV} = -127.753 + 37.388 \ln (W(G))$$

$$\text {MV} = -92.273 + 44.314 \ln (W(G))$$

$$\text {ST} = -13.671 + 11.727 \ln (W(G))$$

### Hyper-Wiener index *HW*(*G*)

*Linear Models:*

$$\text {BP} = 265.904 + 0.106 \cdot HW(G)$$

$$\text {MW} = 143.580 + 0.054 \cdot HW(G)$$

$$\text {EV} = 60.868 + 0.028 \cdot HW(G)$$

$$\text {MV} = 130.630 + 0.033 \cdot HW(G)$$

$$\text {ST} = 46.509 + 0.008 \cdot HW(G)$$

*Quadratic Models:*

$$\text {BP} = 198.310 + 0.233 \cdot HW(G) -0.0000 \cdot HW(G)^2$$

$$\text {MW} = 115.908 + 0.106 \cdot HW(G) -0.0000 \cdot HW(G)^2$$

$$\text {EV} = 46.757 + 0.054 \cdot HW(G) -0.0000 \cdot HW(G)^2$$

$$\text {MV} = 117.672 + 0.058 \cdot HW(G) -0.0000 \cdot HW(G)^2$$

$$\text {ST} = 38.951 + 0.022 \cdot HW(G) -0.0000 \cdot HW(G)^2$$

*Logarithmic Models:*

$$\text {BP} = -444.899 + 122.564 \ln (HW(G))$$

$$\text {MW} = -207.113 + 60.775 \ln (HW(G))$$

$$\text {EV} = -116.990 + 30.865 \ln (HW(G))$$

$$\text {MV} = -83.997 + 37.245 \ln (HW(G))$$

$$\text {ST} = -7.642 + 9.306 \ln (HW(G))$$

### Distance-degree index *DD*(*G*)

*Linear Models:*

$$\text {BP} = 234.079+0.076 \cdot DD(G)$$

$$\text {MW} = 129.050+0.037 \cdot DD(G)$$

$$\text {EV} = 52.833+0.020 \cdot DD(G)$$

$$\text {MV} = 121.769+0.023\cdot DD(G)$$

$$\text {ST} = 44.553+0.006 \cdot DD(G)$$

*Quadratic Models:*

$$\text {BP} = 165.161 + 0.156 \cdot DD(G) -0.0000 \cdot DD(G)^2$$

$$\text {MW} = 104.649 + 0.066 \cdot DD(G) -0.0000 \cdot DD(G)^2$$

$$\text {EV} = 40.882 + 0.034 \cdot DD(G) -0.0000 \cdot DD(G)^2$$

$$\text {MV} = 107.827 + 0.041 \cdot DD(G) -0.0000 \cdot DD(G)^2$$

$$\text {ST} = 38.496 + 0.011 \cdot DD(G) -0.0000 \cdot DD(G)^2$$

*Logarithmic Models:*

$$\text {BP} = -683.669 + 144.565 \ln (DD(G))$$

$$\text {MW} = -320.736 + 70.984 \ln (DD(G))$$

$$\text {EV} = -175.644 + 36.197 \ln (DD(G))$$

$$\text {MV} = -146.997 + 42.626 \ln (DD(G))$$

$$\text {ST} = -29.829 + 11.501 \ln (DD(G))$$

### Gutman index *gut*(*G*)

*Linear Models:*

$$\text {BP} = 241.838 + 0.063 \cdot Gut(G)$$

$$\text {MW} = 132.999 + 0.031 \cdot Gut(G)$$

$$\text {EV} = 54.839 + 0.016 \cdot Gut(G)$$

$$\text {MV} = 126.207 + 0.018 \cdot Gut(G)$$

$$\text {ST} = 43.469 + 0.005 \cdot Gut(G)$$

*Quadratic Models:*

$$\text {BP} = 171.550 + 0.132 \cdot Gut(G) -0.0000 \cdot Gut(G)^2$$

$$\text {MW} = 107.320 + 0.056 \cdot Gut(G) -0.0000 \cdot Gut(G)^2$$

$$\text {EV} = 42.241 + 0.029 \cdot Gut(G) -0.0000 \cdot Gut(G)^2$$

$$\text {MV} = 108.600 + 0.036 \cdot Gut(G) -0.0000 \cdot Gut(G)^2$$

$$\text {ST} = 39.526 + 0.009 \cdot Gut(G) -0.0000 \cdot Gut(G)^2$$

*Logarithmic Models:*

$$\text {BP} = -667.768 + 140.010 \ln (Gut(G))$$

$$\text {MW} = -311.977 + 68.613 \ln (Gut(G))$$

$$\text {EV} = -171.467 + 35.030 \ln (Gut(G))$$

$$\text {MV} = -140.255 + 41.009 \ln (Gut(G))$$

$$\text {ST} = -29.501 + 11.259 \ln (Gut(G))$$

### Harary index *H*(*G*)

*Linear Models:*

$$\text {BP} = 122.938 + 4.970 \cdot H(G)$$

$$\text {MW} = 78.925 + 2.352 \cdot H(G)$$

$$\text {EV} = 26.445 + 1.239 \cdot H(G)$$

$$\text {MV} = 96.393 + 1.354 \cdot H(G)$$

$$\text {ST} = 32.849 + 0.416 \cdot H(G)$$

*Quadratic Models:*

$$\text {BP} = 26.583 + 8.832 \cdot H(G) -0.0344 \cdot H(G)^2$$

$$\text {MW} = 46.503 + 3.621 \cdot H(G) -0.0110 \cdot H(G)^2$$

$$\text {EV} = 13.483 + 1.756 \cdot H(G) -0.0046 \cdot H(G)^2$$

$$\text {MV} = 54.912 + 2.979 \cdot H(G) -0.0140 \cdot H(G)^2$$

$$\text {ST} = 38.228 + 0.206 \cdot H(G) +0.0018 \cdot H(G)^2$$

*Logarithmic Models:*

$$\text {BP} = -609.495 + 255.141 \ln (H(G))$$

$$\text {MW} = -276.121 + 123.058 \ln (H(G))$$

$$\text {EV} = -154.059 + 63.088 \ln (H(G))$$

$$\text {MV} = -112.035 + 71.856 \ln (H(G))$$

$$\text {ST} = -28.451 + 21.399 \ln (H(G))$$

### Additively weighted harary index $H_A(G)$

*Linear Models:*

$$\text {BP} = 139.594 + 0.968 \cdot H_A(G)$$

$$\text {MW} = 87.728 + 0.454 \cdot H_A(G)$$

$$\text {EV} = 30.733 + 0.241 \cdot H_A(G)$$

$$\text {MV} = 102.292 + 0.258 \cdot H_A(G)$$

$$\text {ST} = 33.837 + 0.082 \cdot H_A(G)$$

*Quadratic Models:*

$$\text {BP} = 45.148 + 1.756 \cdot H_A(G) -0.0014 \cdot H_A(G)^2$$

$$\text {MW} = 51.166 + 0.751 \cdot H_A(G) -0.0005 \cdot H_A(G)^2$$

$$\text {EV} = 15.699 + 0.366 \cdot H_A(G) -0.0002 \cdot H_A(G)^2$$

$$\text {MV} = 59.150 + 0.609 \cdot H_A(G) -0.0006 \cdot H_A(G)^2$$

$$\text {ST} = 38.650 + 0.043 \cdot H_A(G) + 0.0001 \cdot H_A(G)^2$$

*Logarithmic Models:*

$$\text {BP} = -916.381 + 238.362 \ln (H_A(G))$$

$$\text {MW} = -422.360 + 114.631 \ln (H_A(G))$$

$$\text {EV} = -229.324 + 58.828 \ln (H_A(G))$$

$$\text {MV} = -194.404 + 66.393 \ln (H_A(G))$$

$$\text {ST} = -56.130 + 20.337 \ln (H_A(G))$$

### Multiplicative weighted harary index $H_M(G)$

*Linear Models:*

$$\text {BP} = 155.477 + 0.753 \cdot H_M(G)$$

$$\text {MW} = 96.026 + 0.350 \cdot H_M(G)$$

$$\text {EV} = \text {{34.812}} + 0.187 \cdot H_M(G)$$

$$\text {MV} = 107.759 + 0.197 \cdot H_M(G)$$

$$\text {ST} = 34.835 + 0.065 \cdot H_M(G)$$

*Quadratic Models:*

$$\text {BP} = 62.076 + 1.402 \cdot H_M(G) -0.0010 \cdot H_M(G)^2$$

$$\text {MW} = 56.319 + 0.617 \cdot H_M(G) -0.0004 \cdot H_M(G)^2$$

$$\text {EV} = 118.011 + 0.303 \cdot H_M(G) -0.0002 \cdot H_M(G)^2$$

$$\text {MV} = 64.130 + 0.491 \cdot H_M(G) -0.0004 \cdot H_M(G)^2$$

$$\text {ST} = 38.771 + 0.039 \cdot H_M(G) +0.0000 \cdot H_M(G)^2$$

*Logarithmic Models:*

$$\text {BP} = -869.745 + 222.717 \ln (H_M(G))$$

$$\text {MW} = -398.486 + 106.843 \ln (H_M(G))$$

$$\text {EV} = -217.253 + 54.868 \ln (H_M(G))$$

$$\text {MV} = -177.889 + 61.416 \ln (H_M(G))$$

$$\text {ST} = -53.887 + 19.301 \ln (H_M(G))$$

## Discussion

This section calculates the statistical parameters for linear, quadratic, and logarithmic models to predict five physical and chemical properties of 15 PAHs, using topological indices as independent variables, see the Tables 4, 5, 6, 7, 8, 9 and 10. The sample size is denoted by “*N*,” with “*a*” as the constant and “*b*” and “*c*” as the coefficients of the independent variables. The correlation coefficient (*R*) indicates the strength and direction of the relationship between two variables. A value of -1 signifies a perfect negative correlation, +1 indicates a perfect positive correlation, and 0 means no correlation. Positive values suggest a direct relationship, while negative values indicate an inverse relationship. In a regression model, a higher $R^2$ value indicates a better fit to the data and stronger explanatory power of the independent variables. Significant *F*-statistic, and *p*-values of regression coefficients below 0.05 confirm the model’s validity. Since the p-values for all models in this experiment were below 0.05, we selected regression models for outcome prediction based on higher $R^2$ values, while simultaneously incorporating diverse types of regression models and topological indices to enhance the generalizability of the experimental findings.

**Table 4:** Statistical parameters for *W*(*G*).

Props.	*N*	*a*	*b*	*c*	*R*	$R^2$	*F*	*p*
*Linear regression model*								
BP	14	0.354	233.484	–	0.964	0.930	159.196	0
MW	15	0.176	128.344	–	0.983	0.966	363.977	0
EV	14	0.091	52.743	–	0.983	0.966	340.476	0
MV	15	0.106	122.658	–	0.967	0.935	188.366	0
ST	15	0.028	43.240	–	0.778	0.605	19.939	0
*Quadratic regression model*								
BP	14	-0.0004	0.740	158.488	0.994	0.987	420.432	0
MW	15	-0.0001	0.313	101.657	0.996	0.993	814.329	0
EV	14	-0.0001	0.161	39.389	0.997	0.995	1075.581	0
MV	15	-0.0001	0.186	106.943	0.980	0.961	146.828	0
ST	15	-0.0000	0.058	37.383	0.799	0.638	10.573	0.002
*Logarithmic regression model*								
BP	14	149.200	-491.673	–	0.994	0.988	983.026	0
MW	15	73.415	-227.300	–	0.991	0.982	713.992	0
EV	14	37.388	-127.753	–	0.989	0.979	552.716	0
MV	15	44.314	-92.273	–	0.980	0.961	319.194	0
ST	15	11.727	-13.671	–	0.790	0.625	21.651	0.001

**Table 5:** Statistical parameters for *HW*(*G*).

Props.	*N*	*a*	*b*	*c*	*R*	$R^2$	*F*	*p*
*Linear regression model*								
BP	14	0.106	265.904	–	0.932	0.868	79.075	0
MW	15	0.054	143.580	–	0.954	0.910	131.847	0
EV	14	0.028	60.868	–	0.954	0.910	121.762	0
MV	15	0.033	130.630	–	0.964	0.930	172.348	0
ST	15	0.008	46.509	–	0.700	0.490	12.484	0.004
*Quadratic regression model*								
BP	14	-0.000	0.233	198.310	0.986	0.973	198.029	0
MW	15	-0.000	0.106	115.908	0.990	0.979	281.5	0
EV	14	-0.000	0.054	46.757	0.992	0.985	361.579	0
MV	15	-0.000	0.058	117.672	0.985	0.970	196.827	0
ST	15	-0.000	0.022	38.951	0.786	0.618	9.707	0.003
*Logarithmic regression model*								
BP	14	122.564	-444.899	–	0.989	0.977	520.498	0
MW	15	60.775	-207.113	–	0.985	0.970	417.977	0
EV	14	30.865	-116.99	–	0.985	0.971	400.193	0
MV	15	37.245	-83.997	–	0.989	0.978	579.541	0
ST	15	9.306	-7.642	–	0.753	0.567	17.020	0.001

**Table 6:** Statistical parameters for *DD*(*G*).

Props.	*N*	*a*	*b*	*c*	*R*	$R^2$	*F*	*p*
*Linear regression model*								
BP	14	0.075	237.591	–	0.965	0.931	162.321	0
MW	15	0.037	130.639	–	0.983	0.967	382.681	0
EV	14	0.019	53.774	–	0.983	0.967	354.321	0
MV	15	0.022	124.439	–	0.960	0.923	154.764	0
ST	15	0.006	43.331	–	0.794	0.631	22.210	0
*Quadratic regression model*								
BP	14	-0.0000	0.156	165.161	0.994	0.988	464.833	0
MW	15	-0.0000	0.066	104.649	0.997	0.995	1124.795	0
EV	14	-0.0000	0.034	40.882	0.998	0.996	1360.696	0
MV	15	-0.0000	0.041	107.827	0.976	0.953	120.937	0
ST	15	-0.0000	0.011	38.496	0.809	0.655	11.369	0.002
*Logarithmic regression model*								
BP	14	144.565	-683.669	–	0.995	0.99	1184.512	0
MW	15	70.984	-320.736	–	0.992	0.984	811.544	0
EV	14	36.197	-175.644	–	0.990	0.979	564.088	0
MV	15	42.626	-146.997	–	0.976	0.953	263.71	0
ST	15	11.501	-29.829	–	0.803	0.644	23.547	0

**Table 7:** Statistical parameters for *Gut*(*G*).

Props.	*N*	*a*	*b*	*c*	*R*	$R^2$	*F*	*p*
*Linear regression model*								
BP	14	0.063	241.838	–	0.965	0.931	160.836	0
MW	15	0.031	132.999	–	0.983	0.967	376.478	0
EV	14	0.016	54.839	–	0.983	0.967	349.031	0
MV	15	0.018	126.207	–	0.953	0.909	129.377	0
ST	15	0.005	43.469	–	0.808	0.653	24.505	0
*Quadratic regression model*								
BP	14	-0.0000	0.132	171.55	0.994	0.989	479.832	0
MW	15	-0.0000	0.056	107.32	0.998	0.996	1374.608	0
EV	14	-0.0000	0.029	42.241	0.998	0.996	1426.597	0
MV	15	-0.0000	0.036	108.6	0.972	0.945	103.779	0
ST	15	-0.0000	0.009	39.526	0.819	0.67	12.206	0.001
*Logarithmic regression model*								
BP	14	140.01	-667.768	–	0.996	0.991	1333.118	0
MW	15	68.613	-311.977	–	0.993	0.985	876.273	0
EV	14	35.03	-171.467	–	0.989	0.979	554.704	0
MV	15	41.009	-140.255	–	0.972	0.945	224.418	0
ST	15	11.259	-29.501	–	0.813	0.662	25.414	0

**Table 8:** Statistical parameters for *H*(*G*).

Props.	*N*	*a*	*b*	*c*	*R*	$R^2$	*F*	*p*
*Linear regression model*								
BP	14	4.97	122.938	–	0.99	0.98	599.791	0
MW	15	2.352	78.925	–	0.994	0.988	1074.391	0
EV	14	1.239	26.445	–	0.991	0.983	676.409	0
MV	15	1.354	96.393	–	0.938	0.88	95.203	0
ST	15	0.416	32.849	–	0.879	0.773	44.191	0
*Quadratic regression model*								
BP	14	-0.0344	8.832	26.583	0.997	0.994	917.251	0
MW	15	-0.011	3.621	46.503	0.997	0.995	1101.482	0
EV	14	-0.0046	1.756	13.483	0.993	0.987	406.353	0
MV	15	-0.014	2.979	54.912	0.953	0.909	59.638	0
ST	15	0.0018	0.206	38.228	0.882	0.777	20.927	0
*Logarithmic regression model*								
BP	14	255.141	-609.495	–	0.995	0.99	1176.466	0
MW	15	123.058	-276.121	–	0.992	0.983	758.732	0
EV	14	63.088	-154.059	–	0.985	0.970	385.700	0
MV	15	71.856	-112.035	–	0.949	0.900	117.185	0
ST	15	21.399	-28.451	–	0.861	0.741	37.261	0

**Table 9:** Statistical parameters for $H_A(G)$.

Props.	*N*	*a*	*b*	*c*	*R*	$R^2$	*F*	*p*
*Linear regression model*								
BP	14	0.968	139.594	–	0.986	0.972	415.884	0
MW	15	0.454	87.728	–	0.989	0.978	575.582	0
EV	14	0.241	30.733	–	0.985	0.971	401.199	0
MV	15	0.258	102.292	–	0.923	0.852	74.544	0
ST	15	0.082	33.837	–	0.896	0.803	53.144	0
*Quadratic regression model*								
BP	14	-0.0014	1.756	45.148	0.995	0.989	503.998	0
MW	15	-0.0005	0.751	51.166	0.994	0.989	537.706	0
EV	14	-0.0002	0.366	15.699	0.989	0.978	246.752	0
MV	15	-0.0006	0.609	59.150	0.945	0.893	49.982	0
ST	15	0.0001	0.043	38.650	0.899	0.808	25.289	0
*Logarithmic regression model*								
BP	14	238.362	-916.381	–	0.992	0.984	726.986	0
MW	15	114.631	-422.36	–	0.988	0.977	547.475	0
EV	14	58.828	-229.324	–	0.980	0.960	289.322	0
MV	15	66.393	-194.404	–	0.938	0.880	95.24	0
ST	15	20.337	-56.130	–	0.876	0.767	42.713	0

**Table 10:** Statistical parameters for $H_M(G)$.

Props.	*N*	*a*	*b*	*c*	*R*	$R^2$	*F*	*p*
*Linear regression model*								
BP	14	0.753	155.477	–	0.980	0.961	292.233	0
MW	15	0.350	96.026	–	0.982	0.965	358.492	0
EV	14	0.187	34.812	–	0.978	0.957	264.356	0
MV	15	0.197	107.759	–	0.907	0.822	60.124	0
ST	15	0.065	34.835	–	0.911	0.830	63.484	0
*Quadratic regression model*								
BP	14	-0.001	1.402	62.076	0.991	0.983	312.129	0
MW	15	-0.0004	0.617	56.319	0.991	0.982	330.438	0
EV	14	-0.0002	0.303	18.011	0.984	0.968	168.916	0
MV	15	-0.0004	0.491	64.130	0.937	0.878	43.121	0
ST	15	0.000	0.039	38.771	0.913	0.834	30.196	0
*Logarithmic regression model*								
BP	14	222.717	-869.745	–	0.988	0.976	485.688	0
MW	15	106.843	-398.486	–	0.984	0.969	406.105	0
EV	14	54.868	-217.253	–	0.974	0.949	223.951	0
MV	15	61.416	-177.889	–	0.927	0.860	79.684	0
ST	15	19.301	-53.887	–	0.888	0.789	48.475	0

**Fig. 2:**
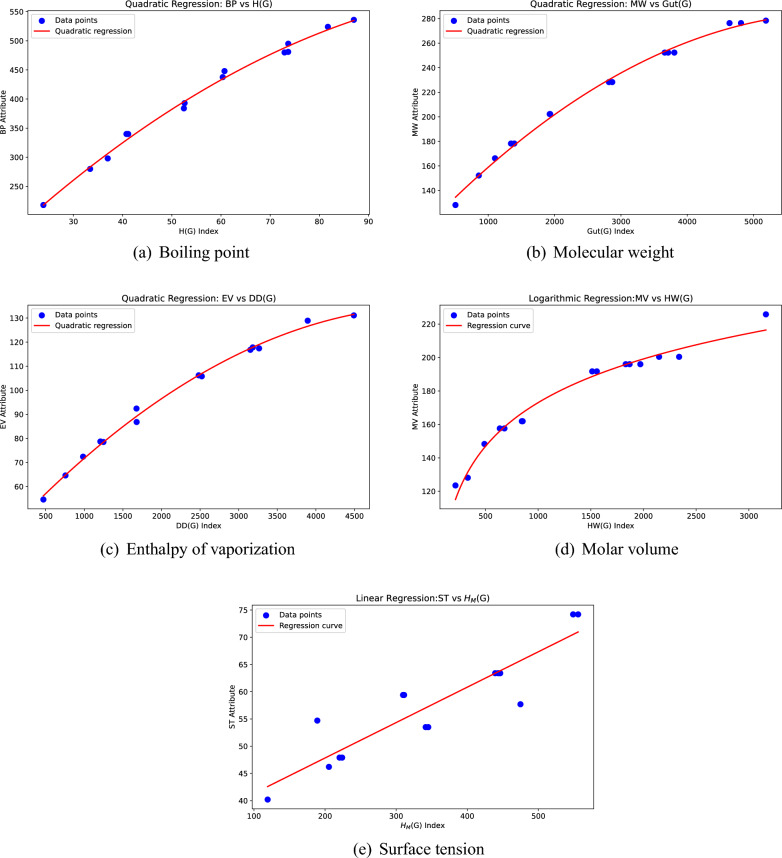
Regression models for different physical properties of PAHs.

1. *To validate the regression models*, we consider five PAHs: Naphthalene, Fluorene, Phenanthrene, Chrysene, and Benzo[a]pyrene. The following regression models are used to calculate the values of the physical properties of PAHs (see Fig. 2):
  Boiling point (modeled using quadratic regression based on the *H* index), with fitting degree $R^2$=0.994.
  Molecular weight (modeled using quadratic regression based on the *Gut* index), with fitting degree: $R^2$=0.996.
  Enthalpy of vaporization (modeled using quadratic regression based on the *DD* index), with fitting degree: $R^2$=0.996.
  Molar volume (modeled using logarithmic regression based on the *HW* index), with fitting degree: $R^2$=0.978.
  Surface tension (modeled using linear regression based on the $H_{M}$ index), with fitting degree: $R^2$=0.830.
2. *To predict the physicochemical properties*, the table below presents the experimental and regression model-calculated values of physicochemical properties for five PAHs: naphthalene, fluorene, phenanthrene, chrysene, and benzo[a]pyrene.
  Boiling point $$= 26.583 + 8.832 \cdot H(G) -0.0344 \cdot H(G)^2$$.
  Molecular weight $$= 107.320 + 0.056 \cdot Gut(G) -0.0000 \cdot Gut(G)^2$$.
  Enthalpy of vaporization $= 40.882 + 0.034 \cdot DD(G) -0.0000 \cdot DD(G)^2$.
  Molar volume $$= -83.997 + 37.245 \ln (HW(G))$$.
  Surface tension $$= 34.835 + 0.065 \cdot H_M(G)$$.

The five physical properties of the five PAHs were computed using the regression models and compared against experimental data, as summarized in Table 11. The model were found to yield accurate predictions for all of the properties mentioned above.

**Table 11:** Comparison of experimental values with the values obtained from regression models.

Properties	Boiling point (BP)	Molecular weight (MW)	Enthalpy of vaporization (EV)	Molar volume (MV)	Surface tension (ST)
Units	$^{\circ }$C at 760 mmHg	g/mol	kJ/mol	$\hbox {cm}^3$	dyn/cm
*Naphthalene*					
Experimental values	218.0	128.17	54.6	123.5	40.2
Values obtained from regression models	218.0	135.60	56.9	116.0	42.6
*Fluorene*					
Experimental values	298.0	166.22	72.4	148.3	46.2
Values obtained from regression models	306.2	168.98	74.3	146.7	48.2
*Phenanthrene*					
Experimental values	340.0	178.23	78.7	157.6	47.9
Values obtained from regression models	331.7	182.47	82.0	156.4	49.4
*Chrysene*					
Experimental values	448.0	228.29	106.2	191.7	53.5
Values obtained from regression models	436.0	265.18	125.3	188.7	57.3
*Benzo[a]pyrene*					
Experimental values	495.0	252.31	117.8	196.0	63.4
Values obtained from regression models	490.6	315.19	149.1	196.6	63.7

## Conclusions

This study investigated the quantitative relationships between molecular topological indices and the physicochemical properties of 15 Polycyclic Aromatic Hydrocarbons (PAHs). The results indicate that the selected topological indices show notable correlations with the target properties, with linear correlation coefficients generally exceeding 0.7. Among the models tested, quadratic regression models based on the Distance-degree index and the Gutman index demonstrated a high degree of fit for predicting molecular weight and enthalpy of vaporization within this dataset, yielding coefficients of determination ($R^2$) above 0.995. Strong correlations were also observed for boiling point and molar volume.These findings contribute to a more quantitative understanding of the structure–property relationships for this specific set of PAHs. The methods explored herein may offer a useful, preliminary tool for estimating key properties in environmental risk assessment and for the initial design stages of PAH-like molecules.

While this study provides insights into the structure–property relationships for a foundational set of 15 polycyclic aromatic hydrocarbons (PAHs), the high performance of these models is confined to the specific dataset and limited descriptor set employed. Their predictive accuracy and generalizability require rigorous validation across a broader chemical space, encompassing diverse classes of environmental pollutants. Therefore, assessing the applicability of this approach to other contaminant families, such as polychlorinated biphenyls (PCBs) or emerging persistent organic pollutants (POPs), and exploring the integration of additional descriptor categories, represent critical steps toward developing a more universally applicable predictive tool.

To address these limitations and enhance the generalizability of our approach, future work will expand the descriptor set by incorporating counting-based indices, such as subtree and subpath numbers^14^. We will investigate whether these additional descriptors can capture complementary aspects of molecular structure, thereby improving predictive accuracy and robustness for a wider array of physicochemical properties across more diverse chemical structures.

## Data Availability

All data generated or analysed during this study are included in this article.
